# Perspective: lessons learned from the COVID-19 pandemic concerning the resilience of the population

**DOI:** 10.1186/s13584-023-00557-w

**Published:** 2023-05-02

**Authors:** Bruria Adini, Shaul Kimhi

**Affiliations:** 1 Department of Emergency and Disaster Management, School of Public Health, Sackler Faculty of Medicine and ResWell Research Collaboration, Tel Aviv University, Tel Aviv, Israel; 2 ResWell Research Collaboration, Tel Aviv University, Tel Aviv, Israel

## Abstract

**Background:**

A vital stakeholder in the successful management of the COVID-19 pandemic is the public. The degree of involvement of the population in managing the pandemic, and the leadership perception of the public, had a direct impact on the resilience of the population and level of adherence to the issued protective measures.

**Main body:**

Resilience refers to the ability to ‘bounce back’ or ‘bounce forward’ following adversity. Resilience facilitates community engagement which is a crucial component of combating the COVID-19 pandemic. The article highlights six insights recognized in studies conducted in Israel during and following the pandemic concerning the resilience of the country’s population. (1) Contrary to varied adversities in which the community serves as an important support system to the individuals, this type of support was substantially impaired during the COVID-19 pandemic, due to the need to maintain isolation, social distancing, and lockdowns. (2) Policy-making during the pandemic should be based on evidence-based data, rather than on assumptions made by decision-makers. This gap led the authorities during the pandemic to adopt measures that were ineffective, such as risk communication based on ‘scare tactics’ concerning the virus, when the highest risk perceived by the public was political instability. (3) Societal resilience is associated with the public’s behavior, such as with vaccine hesitancy and uptake. (4) Factors that affect the levels of resilience include, among others, self-efficacy (impacts individual resilience); social, institutional, and economic aspects as well as well-being (impact community resilience); and hope and trust in the leadership (impact societal resilience). (5) The public should be perceived as an asset in managing the pandemic, thus becoming a vital part of the ‘solution’. This will lead to a better understanding of the needs and expectations of the population and an applicable ‘tailoring’ of the messages that address the public. (6) The gap between science and policymaking must be bridged, to achieve optimal management of the pandemic.

**Conclusions:**

Improving preparedness for future pandemics should be based on a holistic view of all stakeholders, including the public as a valued partner, connectivity between policymakers and scientists, and strengthening the public’s resilience, by enhancing trust in authorities.

## Background

The COVID-19 pandemic impacted most aspects of life in communities worldwide including health, economic, security, societal, and additional facets. Understanding the consequences and impacts not only of the virus itself but also of the management of the pandemic is vital to ensure optimal preparedness and response to the next waves of this pandemic as well as of other emerging or potential communicable diseases. Diverse stakeholders were involved in the management of the pandemic, at both national and international levels, including governance systems, healthcare organizations, economic entities, service providers, pharmaceutical companies, and more. A vital stakeholder in the successful management of the pandemic, though often not recognized as such, is the public. The degree of involvement of the population in designing and implementing the strategies for managing the pandemic, and the perception of the public by each country’s leadership, had a direct impact on the resilience of the population as well as their level of adherence to each of the directives that were issued by the governance systems [27]. Engagement of the public in policy-making during the COVID-19 pandemic has been achieved through varied modes, such as virtual seminars, meetings, academic studies, interviews, or through the social media [3, 20].

## What is ‘resilience’ of the population and why is it a vital component of managing pandemics?

Numerous definitions have been applied to the concept of ‘resilience’ [30], including “the process of effectively negotiating, adapting to, or managing significant sources of stress or trauma” [35], “healthy, adaptive, or integrated positive functioning over time in the aftermath of adversities” [29], or “a dynamic capability which can allow people to thrive on challenges given appropriate social and personal contexts” [16]. Despite the vast diversity of definitions, most definitions consist of three common components: an occurrence of an emergency or dangerous conditions, buffered by protective elements, that lead to a better outcome than anticipated under such circumstances [30]. While in earlier definitions, resilience was believed to be the ability to ‘bounce back’ following adversity, current descriptions refer to the capacity to ‘bounce forward’ after challenging events, resulting in post-traumatic growth rather than distress [7].

Resilience of the population is classified most frequently to three categories: individual, community, and societal resilience. Individual resilience is “the capacity of its members to work together on communal solutions to shocks or adverse circumstances” [4]. Community resilience is “a process linking a set of adaptive capacities to a positive trajectory of functioning and adaptation” [25]. Societal resilience is the ability of groups or societies to “cope with external stresses and disturbances as a result of social, political and environmental change” [1].

Resilience is perceived by most researchers as a dynamic construct, as it may fluctuate among individuals, communities, or societies, over time and circumstances, according to the accessibility and use of assets (competencies or coping capabilities) and resources (protective mechanisms) [13, 26]. During the COVID-19 pandemic, access to both assets and resources was frequently compromised, due to the measures that were decreed by the authorities, such as the regulation to maintain a complete lockdown for prolonged periods. Even the less “extreme” measures, such as the need to maintain social distancing, wearing masks, or home isolation, affected the ability of individuals to seek support from significant others.

Resilience of the population is an important component of managing pandemics as it facilitates the response to difficult challenges and enables adaptation to the changing situation. It enables people, communities, and societies to draw on inner strengths, maintain hope in the face of difficulty, and remain optimistic about the future. Resilience of the population helps to cope with the effects of the pandemic, from social distancing restrictions to economic hardship. It can also reduce feelings of fear and anxiety and help people stay focused on the present instead of projecting their worries into the future. Resilience is not the solution for the pandemic, but it may be a powerful tool to help promote better health outcomes, develop better support systems, and withstand the challenges encountered in all facets of life. Furthermore, resilience is a positive tool for achieving community engagement [28], which is a crucial component of combating the COVID-19 pandemic.

It is vital to identify the lessons that can be learned from the current COVID-19 pandemic. This article focuses on six insights that were recognized during and following the varied waves of the pandemic concerning the resilience of the Israeli population, which should be considered by the different governance systems when planning the response to future pandemics.

## Insight 1: The community is an important support system during adversities, but ‘disappears’ during pandemics

Following varied adversities, it has been extensively demonstrated that the community serves as an essential support system. Following adversities that resulted from both nature-induced events (such as earthquakes, floods, or storms), or human-made conflicts (such as the conflicts or wars in Ukraine or Azerbaijan), it was found that resilience stems mainly from the formal and informal support that the community provides its residents, enhancing their identity, perceived well-being, hope and a sense of fellowship and mutual support [2].

This crucial element of community support was substantially impaired during the management of the COVID-19 pandemic, due to the need for individuals to isolate and distance themselves from the people around them. This frequently led to a lack of support, as physical and emotional connections were broken, and individuals could not physically interact with others in their communities [9, 36]. Furthermore, those that contracted COVID often felt social exclusion, as others feared any contact with them, even after they recovered from the virus [19]. This ‘stigmatization’ was shown to be problematic even beyond the lack of social support, as it led some individuals to avoid testing for COVID or refuse any treatment because they were concerned that it would result in their being shunned from society [6, 11, 12].

Therefore, it is important to find alternative ways of connecting with people during pandemics, such as virtual gatherings, phone calls, online discussions, or other forms of virtual support. Maintaining social connectivity, even remotely, is key to finding comfort in adversity and supporting all individuals, while respecting the required protective measures.

## Insight 2: Don’t assume! look for evidence-based data

When making decisions regarding the response to the pandemic, it is important not to base the response plan on assumptions, but rather on confirmed and updated data. The COVID-19 pandemic has presented that even when it seems that the assumptions are based on solid and well-founded beliefs, the reality that materialized was different. Following are just a few examples that were found during studies that were conducted among the Israeli population during the pandemic.

Throughout the pandemic, the messages relayed to the public were mostly focused on the assumption that the population is concerned with the virus and its potential consequences on health status. In contrast, longitudinal studies have shown that the highest concern of the population throughout the pandemic was the political instability that characterized the Israeli society rather than the health threat [10, 24]. Another misconception was based on the conception that as the elderly population was considered (and accurately so) as the most vulnerable to the virus (along with other populations of special needs), it is also the least resilient sector in society. Conversely, it was shown that older age (≥ 61 years) is associated with lower levels of anxiety and stress, as well as a decreased level of perceived danger, compared to younger populations [21]. The age group that showed the highest levels of distress symptoms, and lower levels of community and societal resilience, was the younger population (aged 31–40 years). Considering that this population is an important pillar of society (as many of them are in mid-career, have children in the school system, etc.) this should impact policymakers when designing measures to combat the pandemic. Another phenomenon to be noted is the comparison of the mean levels of resilience among students in academic institutions to the general public. Whilst many decision-makers believed that students are ‘less impacted’ by the pandemic, studies showed that the mean level of individual resilience among students is lower compared to that of the general population, while their level of distress was higher [10].

Relying on knowledge concerning the characteristics and consequences of the pandemic is instrumental in developing an effective plan of action that contributes to containing and preventing pandemics and/or their outcomes. Therefore, when planning a response to a pandemic, it is important to look for evidence-based data concerning the needs, expectations, and attitudes of the public, that will assist in making informed decisions and facilitate the design of appropriate and relevant response mechanisms.

## Insight 3: Societal resilience is associated with behavior

Societal resilience is used to describe a society's ability to withstand and adapt to various challenges, including the COVID-19 pandemic [34]. Associated behaviors for societal resilience include community organization, communication, collaboration, risk assessment and management, anticipating potential risks, and developing or utilizing existing resources. Not less important is that this type of resilience was found to be associated with attaining the adherence of the population to the measures recommended by the governance systems as part of the campaign to manage the pandemic and resume the full functionality of the Israeli society [5, 23]. This was well demonstrated during the ongoing campaigns that were launched to encourage the population to be vaccinated against COVID-19. Studies have shown that societal resilience is negatively associated with vaccine hesitancy (the higher the societal resilience, the lower vaccine hesitancy) and positively correlated with vaccine uptake (the higher the societal resilience, the higher levels of vaccine uptake, i.e. number of vaccines and/or boosters that were taken) [23]. Trust has been presented as a major component of societal resilience and it impacts on the willingness of individuals to adhere to guidance and directives of the varied authorities. Investing efforts in enhancing the trust of the population in its leadership as well as in strengthening the social integration of all sectors of society, can substantially impact public attitudes and influence their compliance with measures that are recommended by the State’s leadership [11, 12].

## Insight 4: Factors that impact the levels of resilience

The levels of resilience are dependent on several factors that can vary from person to person. These factors can have a significant impact on the resilience of an individual, organization, or system. The COVID-19 pandemic presented a unique opportunity to identify varied factors that impact the resilience of individuals, communities, and society at large. An important insight that should be emphasized is that the levels of resilience are not constant and may vary according to the changed conditions (levels of infectivity, political governance systems, concurrent emergencies, etc.), the length of time of the pandemic itself, as well as the containment measures that are adopted (such as for example, the duration of each lockdown) [21].

The first factor that can affect levels of individual resilience is the level of self-efficacy. Self-efficacy, or the belief in one's ability to handle adversity and act, is an important determinant of resilience. Those who have a higher level of self-efficacy are more likely to be more resilient in the face of the challenging COVID-19 situation [37].

Community resilience during COVID-19 was impacted by the following five main aspects [31]: 1. Social aspects, including a joint identity, social integrity, and effective communication. 2. Institutional aspects, including effective leadership and planning systems. 3. Endurable economic capacities. 4. Functional and accessible vital services. 5. Well-being and quality of life.

The major factors that were found to impact societal resilience included hope, trust in the country’s leadership as well as the social power of finding meaning in life [22, 33].

## Insight 5: The public should not be perceived as the ‘problem’ but rather as the ‘solution’

The COVID-19 pandemic has been a highly stressful period for humanity in communities worldwide. In response, many governments have implemented a variety of social distancing, contact tracing, and other measures to help protect public health. Unfortunately, rather than looking at the general public as an asset in solving this problem, many governments have chosen to blame them for potentially worsening the situation, criticizing their non-adherence to protective measures that were issued, such as being vaccinated, maintaining social distancing, or strictly adhering to lockdowns [8]. The reality is that the public can provide vital solutions to the great challenge COVID presents. People are the most important asset when it comes to slowing the spread of the virus, and their compliance is essential for containing outbreaks and keeping infection rates low. By strictly complying with guidelines from trusted authorities, such as wearing face masks, reducing social contact, or staying at home during isolation, the public can become the most important part of combating the pandemic [32].

To attain such a partnership with the public, it should be perceived as an important stakeholder in fighting the pandemic, rather than a ‘problem’ that should be dealt with. This alternative perception of the public as a key chain in managing any pandemic will lead to a better understanding of the needs and expectations of the population and a more applicable ‘tailoring’ of the messages and guidelines that address the public. The risk communication strategy that was frequently adopted in Israel well reflects this need. For prolonged periods, the messages relayed to the public focused on the risk the virus poses for the life and health of all individuals, and ‘scare tactics’ were used to encourage people to adhere to the protective measures that were relayed by the authorities [15]. Threats of severe fines, the use of the police to enforce adherence to regulations, and the implementation of a “green tag” to differentiate between vaccinated and non-vaccinated people are just some of the measures that were implemented to increase compliance of the population. In contrast, studies have shown that such measures did not substantially increase the adherence of the public to these measures [18]. The elements that were reported as effective in achieving such compliance were rather the understanding that adoption of such measures will safeguard the health and well-being of loved ones or oneself [14]. Perceiving the population as an integral component in designing the appropriate response to the pandemic, adopting and presenting positive and empowering actions that are based on solidarity and mutual responsibility, rather than efforts to instill fear or other deterring measures, prove to be more instrumental in gaining the cooperation and compliance of the public [18]. Recruiting the public’s compliance is dependent on the transparency of actions, sharing information and insights, enhancing trust in the governmental entities and leadership, and addressing the public’s concerns.

## Insight 6: The gap between science and policymaking must be bridged

The substantial effects of the COVID-19 pandemic have demonstrated that science and policymaking must work together to ensure the appropriate management of future waves of the current pandemic as well as other emerging pandemics. Too often the gap between scientific facts and public beliefs or policies does not allow for addressing global health issues effectively. To bridge this gap, a closer collaboration between scientists, public health officials, and policymakers is needed. First and foremost, public policies need to be informed by scientific research and built on evidence-based data. In times of crisis, policymakers must have access to the most updated scientific findings and evidence-based approaches. Pandemics frequently elicit diverse theories of conspiracy concerning their causes and consequences, and scientific findings can be used to refute those that are not based on any founded data [11].

The COVID pandemic highlighted several issues concerning the bridge between science and policymakers. One issue is that many decision-makers were slow to act on scientific advice regarding the perceptions and concerns of the public regarding the pandemic. For example, the assumption in Israel [10] was that the public is mostly concerned with health issues rather than political or economic consequences. This resulted in a focus on the potential damage that can be caused by the virus, leading to a decline in the public’s trust in the leadership and a consecutive decrease in adherence to guidelines issued by the authorities. There was also a lack of public trust in both scientists and politicians, leading to further confusion and mistrust [17]. Furthermore, there was an inadequate flow of information between scientific communities and policymakers, making it difficult for both groups to make informed decisions.

Science offers a wealth of knowledge about the consequences of the pandemic itself and each strategy that is adopted in the effort to successfully manage it, but without proper policy action, this knowledge cannot truly impact. Simultaneously, policymaking that is not based on recognition and in-depth understanding of the needs, expectations, and motivations of the public, cannot be effective and the objectives are thus not met. The lack of an effective bridge between scientific research and governmental decision-making has caused a schism between the two, leading to a suboptimal achievement of ways to best manage the pandemic. Bridging this gap isn’t an impossible feat, as there are a few steps that could be taken by both sides to formulate and ensure effective collaboration. The first step in bridging this divide is for scientists to be better communicators in terms of their findings, publishing them not only in scientific journals but also in reports and publications that are open and accessible to both policymakers and the public at large. At times, there may be disagreements or controversies among varied scientists, but these too should be shared with varied audiences, despite the confusion or frustration that such inconsistencies may cause. Concurrently, policymakers should be aware, updated, and seek scientific findings that will enable them to understand phenomena and trends that characterize and concern the population, and accordingly consider these elements in their decision and policy making.

## Conclusions

The COVID-19 pandemic has provided a unique opportunity to learn lessons concerning the resilience of the population, its impact on the effectiveness of the management strategies that were adopted by decision and policymakers, and ways to improve coping with future pandemics. A major change that is recommended is a holistic view of all stakeholders that should be involved in the management of adversities, including the recruitment of the public as a valued partner, rather than as a ‘problem’ that should be contended with. Enhancing the connectivity between policymakers and scientists and basing the decision-making process on evidence-based data rather than on basic assumptions, is expected to substantially improve the adherence of the population to governmental guidelines and containment measures. Enhancing the resilience of the population, by strengthening trust in the authorities and governance systems, is vital to the successful management of all types of adversities, among them future pandemics.

## Data Availability

Not applicable.
